# From Electrocardiography to the Catheterization Laboratory: A Multimodal Artificial Intelligence Framework for Acute Coronary Syndrome Detection and Risk Stratification

**DOI:** 10.3390/diagnostics16132046

**Published:** 2026-06-30

**Authors:** Marek Tomala, Maciej Kłaczyński

**Affiliations:** 1 Faculty of Medicine and Health Sciences, University of Applied Sciences in Nowy Sącz, 33-300 Nowy Sącz, Poland; 2 Center for Invasive Cardiology, Electrotherapy, and Angiology, Clinical Research Center Intercard, Al. Beliny-Prażmowskiego 60/4, 31-514 Krakow, Poland; 3 Department of Mechanics and Vibroacoustics, Faculty of Mechanical Engineering and Robotics, AGH University of Krakow, Al. Mickiewicza 30, 30-059 Krakow, Poland; maciej.klaczynski@agh.edu.pl

**Keywords:** acute coronary syndrome, artificial intelligence, deep learning, occlusion myocardial infarction, electrocardiography, coronary computed tomography angiography, fractional flow reserve, optical coherence tomography, risk stratification, machine learning

## Abstract

Current acute coronary syndrome (ACS) care relies on sequential, single-modality diagnostics, in which the electrocardiogram, the troponin trajectory, and the coronary angiogram are interpreted independently rather than as a joint signal. This narrative review maps rather than pools the evidence. We selectively searched PubMed, EMBASE, Cochrane CENTRAL, and Web of Science (January 2015–February 2026); study selection was performed by a single reviewer, without duplicate screening, a PRISMA flow diagram, or a formal risk-of-bias assessment. The three key findings are as follows: A machine learning-enabled electrocardiogram (ECG) for diagnosing occlusion due to myocardial infarction achieved an AUC of 0.938 (95% CI = 0.924–0.951) on data not seen during training and correctly diagnosed 42% of patients that expert interpreters missed. A machine learning-enabled high-sensitivity troponin interpretation method, CoDE-ACS, reported an AUC of 0.953 and increased the number of patients ruled out at initial evaluation from 27% to 61%. Angiographically derived physiological methods produced conflicting results—quantitative flow ratios reduced major adverse cardiovascular events (MACE) in the FAVOR III China trial (HR 0.65), but in FAVOR III Europe the angiography-derived approach did not prove non-inferior to FFR; if anything, QFR guidance led to more events (6.7% vs. 4.2%, an event rate about 60% higher in the QFR arm; HR 1.63; 95% CI 1.11–2.41). There was no difference between FFR-angio and FFR in the ALL-RISE trial. These are diagnostic-accuracy and prognostic-association findings; no trial has yet shown that AI-guided ACS care reduces death, reinfarction, or ischemia-driven revascularization.

## 1. Introduction

The evaluation of acute coronary syndrome (ACS), one of the most time-critical assessments in clinical medicine, has not substantially changed over the past two decades. It is performed sequentially in five components: (1) pre-hospital ECG, (2) emergency-department triage, (3) biomarker stratification, (4) invasive angiography and percutaneous coronary intervention (PCI), and (5) post-PCI risk stratification. Each component is evaluated independently; thresholds for advancing from one to the next are determined by population data rather than individual-level data, and little information is shared between them. This fragmentation results in delays in care. Approximately one-third of individuals presenting with an occluded coronary artery do so without ST-elevation myocardial infarction (STEMI) criteria on a standard 12-lead ECG and therefore commonly wait hours before treatment [1,2].

Our argument is simple. AI should be integrated into this pathway as a continuous layer that runs in parallel, supporting clinicians at every step. It can do so in three ways: (1) by automating tasks such as reducing operator-to-operator variability when analyzing ECG waveform morphologies, quantifying coronary angiograms, and defining plaque morphology on OCT/IVUS; (2) by creating a comprehensive composite likelihood score that integrates information from multiple sources including ECG waveforms, troponin concentrations, CT-derived coronary anatomical structure, OCT/IVUS defined plaque morphology, and electronic data entered by the clinician (e.g., medical history); (3) by providing tailored support based upon a previously established risk threshold for each individual patient.

The development of evidence supporting the use of artificial intelligence has been asymmetric across the technology spectrum. Multicenter validation of machine-learning algorithms for interpreting electrocardiograms and estimating troponin concentrations has been performed in large cohorts (thousands of patients). Additionally, there is increasing adoption of AI-enhanced CCTA technology, including photon-counting acquisition and AI-assisted plaque measurement. Evidence demonstrating the benefits of using invasive AI to perform quantitative coronary angiography, measure fractional flow reserve from angiographic data, and identify the location of the culprit lesion in patients undergoing coronary interventions is also emerging. One positive RCT in patients undergoing angiography for stable coronary disease or acute coronary syndromes beyond the acute phase was recently published (FAVOR III China) [3]; another failed to show non-inferiority of an angiography-derived approach against pressure wire-guided FFR (FAVOR III Europe) [4]; and another, enrolling predominantly stable coronary disease with STEMI excluded, demonstrated non-inferiority to pressure wire-guided FFR (ALL-RISE) [5].

AI applied to intracoronary imaging to analyze plaque morphology has demonstrated good discriminative ability in identifying patients at high risk for future cardiovascular events due to vulnerable plaques, such as thin-cap fibroatheromas. However, AI-IVUS evidence specifically is predominantly procedural rather than event-related. Regardless of the modality used, there is a consistent disconnect between the demonstration of diagnostic accuracy in AI-generated results and the demonstration of outcome-level validation of those results.

The purpose of this article is to review existing literature supporting the use of AI in the management of ACS. Specifically, we have divided our discussion into six distinct areas: AI-ECG (Section 3), Machine Learning Biomarkers (Section 4), AI-CCTA (Section 5), Angiography-Derived Physiology and Angiography-Based Invasive Interventional Technologies (Section 6), Intracoronary Imaging-Based AI Technologies (Section 7), and Multimodal Integration and Cross-Modality Risk Stratification (Section 8). Following these sections, we discuss Implementation/Regulation/Equity/Limitations (Section 9 and Section 10) and Future Research Directions (Section 11), and conclude with our Conclusions, in which observational findings are distinguished from recommendation statements (Section 12).

**Figure 1:**
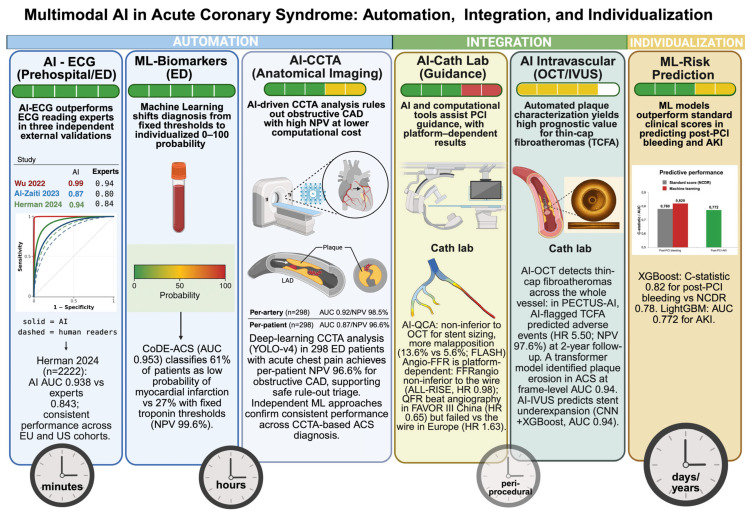
Multimodal artificial intelligence (AI) across the acute coronary syndrome (ACS) pathway. Artificial intelligence is used in different ways at different stages of a patient’s experience with acute coronary syndrome. Three types of roles have been identified, as follows. Automation: pre-hospital and emergency department triage using AI-assisted electrocardiography (ECG); machine-learning interpretation of cardiac biomarker results; coronary computed tomography angiography (CCTA) with AI assistance to rule out obstructive disease. Integration: cath-lab guidance via AI-based quantitative coronary angiography and angiography-derived physiology; intracoronary imaging (optical coherence tomography [OCT] and intravascular ultrasound [IVUS]) for thin-cap fibroatheroma, plaque erosion, and stent under-expansion. Individualization: machine-learning models for post-PCI bleeding and acute kidney injury. Colored bars indicate the relative maturity of evidence in each domain. Clocks indicate the time horizon of operative activities (minutes, days, years). Full statistics and references can be found in the corresponding sections. In the AI-ECG panel, receiver operating characteristic curves are drawn as solid lines for the AI models and as dashed lines for human readers, with curve colors matching each study (Wu 2022 [6], red; Herman 2024 [7], green; Al-Zaiti 2023 [1], blue). In the ML-Risk panel, bars show predictive performance: gray, standard NCDR score; red, machine learning for post-PCI bleeding; green, machine learning for post-PCI acute kidney injury. The horizontal green-to-red bar denotes the probability scale (0–100), and arrows indicate the diagnostic step highlighted in each panel. The studies summarized in this figure are reported in the references [1,3,4,5,6,7,8,9,10,11,12,13,14,15,16]. Abbreviations: ACS = acute coronary syndrome; AI = artificial intelligence; CCTA = coronary computed tomography angiography; ECG = electrocardiography; IVUS = intravascular ultrasound; OCT = optical coherence tomography; PCI = percutaneous coronary intervention.

Figure 1 maps this structure. The six sections that follow correspond to the six panels, arranged left to right along the clinical timeline of acute coronary syndrome, from a prehospital electrocardiogram read in minutes to risk prediction that unfolds over days to years. Reading across the panels also recovers the three roles set out above: automation at the front of the pathway, multimodal integration in the catheterization laboratory, and individualized risk prediction at the end.

This review differs significantly from previous narrative syntheses focused on AI utilization in multiple ways. Most importantly, few prior reviews synthesize evidence from six AI modalities (electrocardiogram interpretation, biomarkers, CCTA, angiography-derived physiology, intracoronary imaging, and multimodal risk prediction) within a single framework; most have addressed one modality at a time. This review also addresses three clinically actionable questions: Can AI-ECG rescue occlusion MIs identified after STEMI criteria fail? Can AI-CCTA safely delay invasive catheterization in patients considered low- to moderate-risk? Is Angiography-Derived Fractional Flow Reserve Non-Inferior to Pressure-Wire Guidance? Finally, this review focuses solely on adults with ACS, in which the translation between diagnosis and outcome is most critical for clinical decision-making.

## 2. Materials and Methods

We conducted a non-systematic narrative review designed to map the evidence rather than estimate pooled effects. We searched four databases (PubMed/MEDLINE, EMBASE via OVID, the Cochrane Central Register of Controlled Trials, and Web of Science), combining three concept blocks: (a) acute coronary syndromes (and related diagnoses such as STEMI, NSTE-ACS, OMI, MINOCA, unstable angina, and acute MI); (b) artificial intelligence, machine learning, and deep learning, named platforms (PMcardio, Queen of Hearts, eOMI, ECG-SMART, CoDE-ACS, HeartFlow, FFR-angio, QFR, and Ultreon), and named architectures (convolutional neural networks, transformers, gradient boosting); and (c) clinical application areas (ECG, troponin, CCTA, FFR, angiography, OCT, IVUS, risk stratification). In addition to these sources, the investigators reviewed citations referencing previous seminal articles. The inclusion criteria were: (1) peer-reviewed publication; (2) original research; (3) systematic review, protocol, or registry record; (4) a minimum of 100 patient participants; (5) a suspected or confirmed acute coronary syndrome population; (6) external validation preferable; and (7) an interpretable performance metric (area under the curve with 95% confidence interval, sensitivity, specificity, hazard ratio, or negative predictive value). Investigators excluded conference abstracts subsequently published in full, editorials, pediatric populations, post-CABG patients, and studies that used AI for asymptomatic screening. Studies that used high-definition IVUS data were included when available; the sparse dedicated AI literature in this area is itself a finding, addressed in Section 7. The reviewers did not generate a PRISMA flow diagram or conduct a formal risk-of-bias assessment because this was a narrative review, not a systematic review. A single reviewer carried out study selection and data extraction without duplicate screening or inter-rater agreement; the search was therefore selective and scoping in nature, and the inclusion criteria listed above guided rather than protocolized that selection. For each pair of trials reporting opposing findings (for example, angiography-derived fractional flow reserve), the reviewers document the methodological differences between the two studies. All claims regarding quantitative AI on ECG-derived data were manually extracted from the source articles and cross-checked for accuracy. Claims from AI-CCTA were based on a secondary database created specifically for verification. The reviewers adhered to the SANRA quality standards for narrative reviews [17] and cited the most informative subset of studies. A future systematic review with a registered protocol and formal risk-of-bias adjudication would be desirable, particularly for AI-ECG detection of OMI, AI-CCTA in suspected NSTE-ACS, and machine-learning troponin algorithms, which now have enough mature evidence to support quantitative synthesis. Because the cited studies differ in population, reference standard, validation strategy, endpoint, and intended use, their performance metrics (AUC, sensitivity, specificity, negative predictive value, and hazard ratios) are not directly comparable across modalities; and should be read in place of any head-to-head metric ranking.

## 3. AI-Enabled Electrocardiography for ACS Detection and Occlusion Myocardial Infarction Identification

### 3.1. The STEMI/OMI Diagnostic Problem

The ECG is the only test in the acute chest pain pathway that provides a diagnostic signal within seconds of patient contact. STEMI criteria, set in the Fourth Universal Definition of Myocardial Infarction, were calibrated to identify ST-segment elevation at the J-point in two contiguous leads, with sex- and age-specific cutoffs in V2/V3 [18]. The criteria perform well in terms of specificity, but were never designed to detect total or near-total coronary occlusion that fails to produce ST elevation on the index recording. Aslanger and colleagues have argued that this is the core flaw of the STEMI/NSTEMI dichotomy: in the general acute MI population, approximately one-third of acute coronary occlusions present without STE on the index ECG and are coded as NSTEMI; the DIFOCCULT-3 trial is now prospectively quantifying this gap [19]. In the Meyers 2021 series, only 60% of OMIs (67 of 108) met STEMI criteria [2]. The occlusion myocardial infarction (OMI) framework recasts the question. The substrate is the lesion, not the waveform; the diagnostic tool is whatever detects the lesion fastest.

### 3.2. Modeling Strategies for AI-ECG

AI-ECG has done the most work on that question. Al-Zaiti’s ECG-SMART takes the engineered-features route, using 73 morpho-temporal descriptors, a random forest classifier, and treeSHAP for feature-level attribution [1]. Herman’s group went in the opposite direction, using raw waveforms and an end-to-end deep convolutional network, eOMI v1 [7]—and then recalibrated it for higher specificity, which was sold commercially as aOMI v1/Queen of Hearts (Powerful Medical, Bratislava, Slovakia) [20]. Cho and colleagues’ DeepECG-AMI sits somewhere in between architecturally but is methodologically further out, with a vision transformer pretrained via self-supervision on roughly a million unlabeled ECGs [21]. The three are not interchangeable. They were trained on different cohorts, validated against different reference standards, and report different operating points, which is what makes a side-by-side reading worthwhile and what the rest of this section attempts to do.

### 3.3. Engineered-Feature Classifiers—ECG-SMART

Al-Zaiti et al. [1] created ECG-SMART, a random forest classifier trained on 73 morpho-temporal characteristics selected by lasso regression and recursive feature elimination from 554 candidate features, with Tree SHAP for interpretability. With an independent test group of 3287 patients who were in emergency medical services before hospitalization in Orange County, CA, and Charlotte-Mecklenburg County, NC, they found that their algorithm had an AUC of 0.87 (95% CI, 0.85–0.90) while the AUC for practicing physicians was 0.80 (95% CI, 0.77–0.83) and the AUC for Philips DXL was 0.75 (95% CI, 0.71–0.79). The combined sensitivity and specificity of the ECG-SMART algorithm were 86% (CI: 81–91%) and 98% (CI: 97–99%), respectively. The total missed-event rate for the ECG-SMART algorithm was 0.9% (*n* = 28/3287). The most highly ranked SHAP values included: ST amplitude at J + 80 ms in lead III (#1), aVL (#2), and V2 (#5); T amplitude in aVL (#3); and the ratio of principal component one of ST variance and T variance across all leads (#4). Since there are reciprocal changes in inferior occlusion that occur early in lead aVL and are also identified at rank positions #2 and #3, these SHAP rankings validate a basic principle that bedside clinicians have utilized for many years. Both the ECG-SMART AUC reported here and the eOMI AUC reported below were derived from cohorts enriched for OMI; at the lower OMI prevalence of unselected chest-pain populations, the corresponding positive predictive value falls, so these discrimination figures should be read with this prevalence dependence in mind.

### 3.4. End-to-End Deep Convolutional Networks—eOMI v1/Queen of Hearts

Herman et al. [7] created eOMI v1, a completely automated, end-to-end deep convolutional neural network of approximately 210,000 parameters (approximately 60,000 across the two convolutional layers and six residual blocks for feature extraction, and approximately 150,000 in the two fully connected layers for classification) that can take a raw 12-lead waveform as input and generate a continuous OMI score ranging from 0 to 1. Based on receiver operating characteristic (ROC) analysis, two operating-point thresholds were selected: 0.1106 as the primary threshold and 0.5995 to replicate STEMI specificity. On a test dataset of 2222 patients (3254 ECGs; 2263 contacts; OMI prevalence 21.6%), eOMI had an AUROC of 0.938 (95% CI 0.924–0.951), significantly greater than that of ECG specialists (0.843; 0.821–0.864) and STEMI criteria (0.651; 0.629–0.672) (both *p* < 0.01). At the primary threshold of 0.1106, sensitivity for eOMI was 80.6% (0.768–0.840) versus 73.0% for ECG specialists and 32.5% for STEMI criteria; specificity for eOMI was 93.7% (0.926–0.948) versus 95.7% and 97.7%, respectively; accuracy was 90.9% (0.897–0.920). The practical finding was the missed OMIs. Of the 330 cases missed by STEMI criteria, 112 (33.9%) were revascularized within two hours, and of the 218 patients who underwent delayed catheterization, the AI identified 133 (61%) on the first ECG; of the OMIs missed by ECG specialists, the AI rescued 56 (42%), with 58.9% being inferior/posterior culprits. Under AI guidance, mean time-to-OMI-diagnosis decreased from 5.3 h to 2.3 h (*p* < 0.001), a decrease of approximately three hours [7]. The subsequent aOMI v1/Queen of Hearts model [20] examined a different question: among patients undergoing emergent cath lab activation for suspected STEMI, how effectively does AI identify true STEMI (including equivalents such as isolated posterior MI, hyperacute T waves, and the De Winter pattern) from STEMI mimics? True STEMI occurred in 58.2% (601/1032) of patients treated at the three U.S. hospitals involved in this study (Beth Israel Deaconess Medical Center, University of California-Davis Medical Center, Memorial Hermann-Texas Medical Center; January 2020–May 2024), with good discrimination (AUC 0.94; 95% CI 0.92–0.95) [20]. At the on-label threshold of 0.50, the model correctly classified 87.4% of cases, with a sensitivity 92.0% (89.7–94.1%); specificity 81.0% (77.2–84.5%); false-positive activation rate 7.9% (6.4–9.6%); positive predictive value 87.1% (84.5–89.6%); negative predictive value 87.9%. Both sensitivity and false-positive activation rate exceeded those of standard care (sensitivity 0.710; 0.674–0.746; false-positive activation rate 0.418; 0.389–0.447). A reclassification analysis with a threshold of 0.05 gave PPV 0.768, NPV 0.905, and a false-positive activation rate of 0.168. We specifically note that 16.8% is the false-positive activation rate, not the PPV, as it has sometimes been mislabeled in secondary reporting. Of the 39.6% (238/601) of true STEMI cases that did not meet conventional STEMI criteria on their index ECG (STEMI equivalents), the AI model’s sensitivity was 83.6%, versus 58.0% for standard-of-care triage (*p* < 0.01). The model also improved the recategorization of biomarker-based false positives identified as STEMI (biomarker-negative false positives: 91% [277/306]; benign early repolarization: 93%; nonspecific ST/T changes: 92%). However, 33.2% (79/238) of initial STEMI-equivalent cases ultimately evolved into conventionally defined STEMI on serial ECGs, and 73.5% (442/601) of all true STEMI cases eventually evolved into conventionally defined STEMI before angiographic confirmation. These data reflect better timing of diagnosis rather than improved case detection [20]. Independent external validation of the Queen of Hearts model was performed by Sharkey et al. [22] in a multicenter U.S. suspected-STEMI registry, in which the model demonstrated an AUROC of 0.952 (95% CI 0.924–0.966) and correctly identified 93.8% of confirmed AMI culprit lesions as OMI-positive.

### 3.5. Large-Scale Hybrid Models—DeepECG-AMI

Cho and colleagues [21] reported the largest external validation to date for any AI-ECG model. The Chonnam derivation cohort comprised 300,627 patients (723,389 ECGs; AMI prevalence: 1.95%); external validation was extended to 259,454 patients. The architecture is a vision transformer with 12 transformer blocks, pre-trained in a self-supervised manner on 1,028,938 unlabeled ECGs. External AUC reached 0.968 (95% CI, 0.965–0.971) for AMI requiring revascularization overall, with subgroup AUCs of 0.991 (0.989–0.993) for STEMI and 0.947 (0.942–0.952) for NSTEMI [21]. The masking analysis is conceptually instructive: when the QRS complex is masked, AUC degradation is 0.044 for STEMI and 0.107 for NSTEMI; when the T wave is masked, degradation is 0.043 for STEMI and 0.070 for NSTEMI. In STEMI, the QRS and T-wave contribute approximately equally; in NSTEMI, the QRS dominates by roughly 1.5-fold, consistent with the clinical intuition that subtle QRS changes (R-wave loss, terminal S-wave attenuation, Q-wave progression) carry diagnostic weight beyond ST-T morphology when the ST segment is non-diagnostic. Operating points at sensitivity fixed at 0.9 in the external set yielded for AMI: sensitivity 0.884 (0.873–0.896), specificity 0.929 (0.928–0.930), PPV 0.129 (0.125–0.134), NPV 0.999 (0.998–0.999); the low PPV reflects the extreme imbalance of the external set (0.42% AMI prevalence) and is expected mathematically rather than a defect of discrimination. Prevalence drives PPV; AUC does not move.

### 3.6. Culprit-Vessel Identification

The limited analysis of culprit-vessel determination demonstrates the capabilities and limitations of CNN architectures. Wu et al. [6] used a two-stage CNN-LSTM architecture on 12-lead ECG data. Stage one identified whether an electrocardiogram indicated STEMI or Control. Two separate models were created within stage two. Model 1 analyzed whether the left anterior descending artery (LAD) had been affected by the myocardial infarction, compared to the right coronary artery-left circumflex (RCA-LCX). Model 2 determined whether the right coronary artery (RCA) or the left circumflex (LCX) had been damaged. When they evaluated their CNN model’s ability to determine whether the LAD or RCA/LCX arteries had been damaged, the AUC values were 0.96 and 0.81, respectively. The CNN-LSTM model demonstrated reduced performance for the RCA-versus-LCX distinction, with an AUC of 0.81. CNNs demonstrate diminished ability to distinguish whether a coronary occlusion occurred in the posterior or non-posterior territory. These findings are consistent with prior research demonstrating that electrocardiograms can determine when a coronary occlusion has occurred, but cannot provide conclusive evidence of whether it occurs in a posterior or non-posterior territory.

### 3.7. Foundational Architectures and Manual-Criterion Anchors

We document our methodology and provide references to those seminal papers that established the foundational literature within the domain. Hannun and colleagues used a 34-layer deep neural network to assess 91,232 single-lead ambulatory ECGs from 53,549 people. Their method achieved an average F1 score of 0.837 across 12 different rhythm classes, far superior to the mean performance of cardiologists (0.780) [23]. In the PTB-XL benchmark (Strodthoff et al.), xresnet1d101 outperformed each of its recurrent counterparts in classifying 71 ECG labels across 21,837 records [24]. Chen and colleagues applied ResNet to PTB-XL data and assessed it using the Chapman-Shaoxing external dataset (*n* = 205, 20% AMI prevalence); ResNet had a test area under the ROC curve of 0.977 (95% CI 0.961–0.991), with area-under-the-curve (AUC) values ranging from 0.961 (anterior MI) to 0.996 (anterolateral MI) [25]. None are considered AI-OMI articles; together, however, they demonstrate that deep learning on raw 12-lead waveforms can classify patterns as well as humans do.

Meyers et al. [26] is not an AI article but one that validates manual ECG criteria for diagnosing OMI (STDmaxV1–4, the maximal ST-segment depression in leads V1–V4). Two independent expert observers achieved a kappa of 0.893. Sensitivity was 37.4%. Specificity was 97.6%. The positive likelihood ratio was 15.60. The high asymmetry of this result suggests that the STDmaxV1–4 ‘rule-in’ criterion may prove useful in confirming posterior OMI when positive but fails to diagnose approximately two-thirds of OMI events when negative. We therefore treat this as the upper limit of what is achievable by human observers without AI for diagnosing OMI; any AI diagnostic claim for OMI via ECG must demonstrate comparable or improved performance relative to trained human observers using ECG-based rules.

There are two limitations to consider regarding this approach. First, the operating point for an AI-ECG will depend on whether it is used for prehospital or cath-lab activation. For example, if an AI system gives a false-negative result in a patient with an acute inferior wall MI, it will likely cause harm because treatment is delayed; conversely, if it gives a false-positive result in a patient with chest pain from other causes, it causes little harm because treatment is initiated unnecessarily. Both Herman models [7,20] were designed to account for these trade-offs between sensitivity and specificity: the primary cutoff of 0.1106 [7] for the eOMI v1 model was optimized for prehospital/ED settings, whereas the on-label cutoff of 0.5 [20] for the aOMI/QoH model was optimized for cath-lab activation. As shown in Table 1, the false-positive activation rate fell to 7.9% with the on-label cutoff 0.50, compared with the 41.8% currently observed with standard-of-care methods. The reclassification threshold of 0.05 [12], with its associated false-positive activation rate of 16.8%, represents the platform’s rule-out anchor.

**Figure 2:**
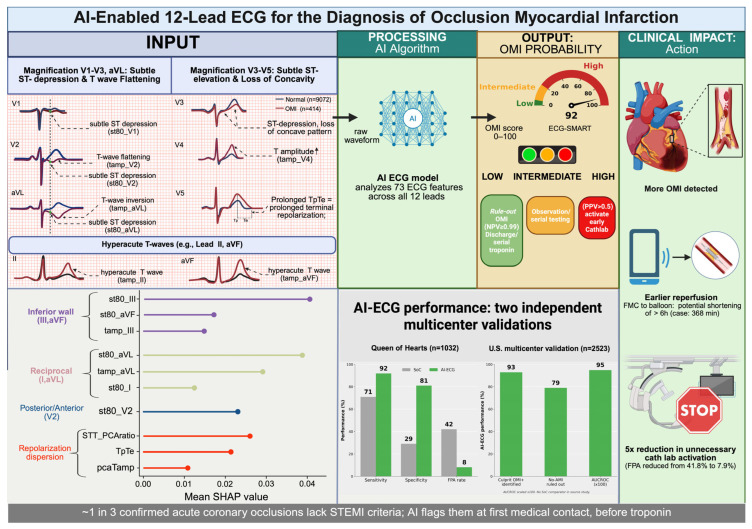
Artificial intelligence (AI)-enabled 12-lead electrocardiography for the diagnosis of myocardial infarction (MI). Input: magnified complexes show the subthreshold pattern of OMI (slight ST-segment depression, T-wave flattening, loss of ST-segment concavity, hyperacute T-wave); median beats from 414 OMI patients and 9072 controls. Processing: the model analyzes 73 features across all 12 leads. Output: a calibrated OMI score (0–100) assists triage: rule out (NPV ≥ 0.99), observation, or cath-lab activation (PPV > 0.5). Clinical impact: earlier diagnosis at initial medical contact, before troponin elevation, resulting in fewer unnecessary activations. The SHAP panel highlights the features that drive the ECG-SMART score, as reported by Al-Zaiti et al. [1]. In validation, Herman et al. [20] (*n* = 1032) increased index-ECG sensitivity to 92.0% (vs. 71.0%) and decreased false-positive activations from 41.8% to 7.9%; Sharkey et al. [22] (*n* = 2523) reported an AUC of 0.952. About one-third of acute coronary occlusions identified via angiography do not fulfill STEMI criteria [19]. In the magnified complexes, blue tracings denote normal beats (*n* = 9072) and red tracings denote OMI beats (*n* = 414); dashed lines mark the J-point + 80 ms measurement, and arrows indicate the labeled ECG features. In the SHAP plot, color groups by territory (inferolateral, anterolateral, antero-posterolateral, repolarization dispersion). The traffic-light icon encodes low (green), intermediate (amber), and high (red) OMI probability; gray and green bars denote standard of care and AI-ECG, respectively. AUC, area under the receiver operating characteristic curve; NPV/PPV, negative/positive predictive value; SHAP, Shapley additive explanation; STEMI, ST-elevation myocardial infarction.

Second, AI-ECG is an aid to decision-making at the level of individual contact rather than at the level of the individual person. Future research may integrate multiple consecutive ECG assessments, dynamic trends, and/or multi-lead Holter monitoring, although none has been tested in a multicenter trial. The AI-ECG pathway for occlusion myocardial infarction, from subthreshold ECG features through a calibrated OMI probability to the triage decision it supports, is summarized in Figure 2.

## 4. Machine Learning for Cardiac Biomarkers

Troponin remains the biochemical anchor for the diagnosis of ACS. The challenge with using a single, binary cutoff at the 99th percentile of the distribution of Troponin values is that it will treat very different patients similarly. In particular, the implications of having a Troponin value 1.5 times above the upper limits of normal (ULN) are quite different as regards the likelihood of Type I MI across age and sex; e.g., much higher risk in young men presenting acutely with chest pain than in older women with CKD. Three machine-learning methods can recover the continuum lost once a threshold is applied to hs-cTnI values. The CoDE-ACS algorithm employs gradient boosting to generate a continuous probability of type 1 MI (0–100) from the hs-cTnI value at initial evaluation and/or at multiple post-presentation intervals, incorporating age, sex, comorbid conditions, and hours since symptom onset [16]. It was tested against 10,038 patients in a derivation cohort (48% female) and 10,286 patients in an external validation set from seven cohorts, yielding an area under the curve (AUC) of 0.953 (95% CI, 0.947–0.958) [16]. The triage implications of CoDE-ACS are apparent: 61% of patients presenting to emergency departments were assigned a low-probability classification, with an NPV of 99.6%, compared with 27% assigned a low probability using fixed troponin thresholds. Thirty-day cardiac mortality was 0.1% in the low-probability group, 0.5% in the intermediate group, and 1.8% in the high-probability group; these differences continued through one year (*p* < 0.001: low 0.3%, intermediate 2.8%, high 4.2%). CoDE-ACS classified fewer patients as high-probability than fixed thresholds (10% vs. 16%); however, the positive predictive value for high-risk classification was greater (75.5% vs. 63.6%), indicating fewer false-positive rule-ins [16]. The Myocardial Ischemic Injury Index (MI3) used a smaller feature set (sex, age, paired hs-cTnI values) and was developed on 3013 patients and validated on 7998. It achieved an AUC of 0.963 (95% CI, 0.956–0.971) for type 1 MI. At MI3 < 1.6, 69.5% of patients were low-risk (NPV 99.7%, 95% CI 99.5–99.8; sensitivity 97.8%); at ≥49.7, 10.6% were high-risk (PPV 71.8%) [27]. Other comparators performed poorly: the ESC 0/3-h pathway had a sensitivity of 82.5% and specificity of 92.2%, and the single point-in-time 99th-percentile cutoff had a sensitivity of 89.6% and specificity of 89.3% [27]. The lesson is that the slope of the troponin rise or fall matters more than the absolute level: a flat troponin over three hours behaves differently from a rising one, even when both are near the same threshold. ARTEMIS-POC [28] extends this concept to a single point-of-care (POC) hs-cTnI measurement, addressing the shorter turnaround time of POC assays compared with central laboratory assays. ARTEMIS-POC was validated across two cohorts (US and AU; *n* = 2560) and provided safe rule-out in 35.1% of patients at presentation, with an NPV of 99.96% (95% CI, 99.64–99.96) and a sensitivity of 99.68% (95% CI, 97.21–99.70). Safe rule-out rates for the standard ESC and ACC 0-h algorithms were significantly lower (15.2% and 13.8%, respectively). No type 1 MIs were missed; all missed events were type 2. Where central-laboratory hs-cTnI is unavailable, ARTEMIS-POC provides a significant operational improvement [28]. Each of these three systems has three structural limitations. First, training-set composition: CoDE-ACS, MI3, and ARTEMIS-POC were developed primarily in Caucasian European, Australian, or North American groups, with no comparable analyses in South Asian, sub-Saharan African, or East Asian populations. Second, sex-specific calibration is incomplete: Wenzl et al. showed that the GRACE 2.0 score with uniform cut points discriminated in-hospital mortality less well in women than in men (AUC 0.82 vs. 0.86; *p* < 0.0001) and redeveloped it as the sex-specific GRACE 3.0 score [29]. Third, high-sensitivity troponin I assays report values on the same scale; those scales are not directly convertible, so a model trained on one vendor’s assay generally requires recalibration before use with another vendor’s assay. Multimodal fusion (ECG plus troponin) shows early promise: Liu and colleagues reported that a combined ECG-plus-troponin model achieved an AUC of 0.978 for NSTEMI detection, exceeding deep learning on the ECG alone (0.877) and troponin alone (0.950); however, the study was single-center and retrospective and needs prospective external validation [30]. The three systems suit different settings. CoDE-ACS [16] fits emergency departments with central-laboratory hs-cTnI testing, where a continuously generated ACS probability adds decision support; MI3 is similar but more portable across hospital systems, given its smaller feature set [27]; and ARTEMIS-POC fills the gap in community EDs or prehospital settings with high-sensitivity troponin, where rapid rule-out at first contact is the priority [28]. No prospective trial has compared these algorithms on a common dataset, so the choice depends on operational setting and resources rather than proven superiority. The 2023 ESC ACS guideline has already recognized this trend, stating that artificial intelligence models using serial high-sensitivity troponin levels together with individual risk profiles “have been proposed to be useful” for personalized diagnosis of suspected MI, but it makes no formal recommendation, assigns no Class or Level of Evidence to these tools, and addresses no AI-ECG application, thus highlighting the gap between the emerging evidence reviewed above and current guideline endorsement [31].

## 5. AI-Enhanced Coronary CT Angiography

The 2023 ESC ACS guideline [31] gives CCTA a Class IIa recommendation for patients with no electrocardiogram (ECG) changes and inconclusive high-sensitivity troponin, while the 2021 AHA/ACC chest pain guideline [32] gives CCTA a Class 1 recommendation for the exclusion of atherosclerotic plaque and obstructive coronary artery disease in intermediate-risk patients with no known CAD whose initial emergency department evaluation for ACS was negative or inconclusive. As mentioned earlier, what has been lacking until now is the ability to use computerized algorithms to convert standard CCTAs into quantifiable diagnostic instruments. That layer now exists in three forms: (1) artificial intelligence-assisted quantitative computed tomography (AI-QCT); (2) artificial intelligence-based CT-derived fractional flow reserve (FFR-CT); and (3) artificial intelligence-based plaque phenotyping.

**Figure 3:**
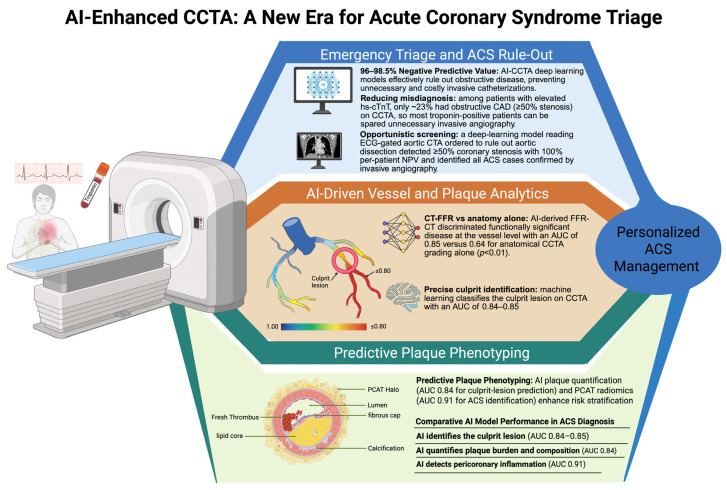
AI-enhanced coronary CT angiography (CCTA) across acute coronary syndrome (ACS) triage, in three layers. Emergency rule-out: deep-learning CCTA rules out obstructive disease with high negative predictive value. Vessel and plaque analytics: AI-derived CT fractional flow reserve (FFR-CT) outperforms anatomical grading at the vessel level (AUC 0.85 vs. 0.64), and machine learning identifies the culprit lesion (AUC 0.84–0.85). Predictive plaque phenotyping: AI plaque quantification predicts culprit lesions (AUC 0.84), and pericoronary adipose tissue (PCAT) radiomics identifies ACS. Values are illustrative of the cited studies; full statistics appear in Section 5. The three colored bands correspond to the triage layers (blue: emergency rule-out; orange, vessel and plaque analytics; green, predictive plaque phenotyping). On the coronary tree, the color scale runs from blue (FFR-CT 1.00) to red (≤0.80), and the arrow marks the culprit lesion. Leader lines label the plaque components (lumen, fibrous cap, lipid core, fresh thrombus, calcification, and pericoronary adipose tissue halo). The studies summarized in this figure are reported in the references [14,33,34,35,36,37,38,39]. Abbreviations: ACS, acute coronary syndrome; AI, artificial intelligence; AUC, area under the curve; CCTA, coronary computed tomography angiography; FFR-CT, CT-derived fractional flow reserve; PCAT, pericoronary adipose tissue.

These three layers, spanning emergency rule-out, vessel- and lesion-level functional analysis, and predictive plaque phenotyping, are summarized in Figure 3.

### 5.1. Stenosis Detection and Culprit-Lesion Identification

Kim et al. reported the only deep-learning stenosis-detection model tested specifically in an emergency department population with acute chest pain [14]. The approach utilized YOLOv4 (an object detector utilizing a CSPDarknet53 backbone) on curved MPRs. The model was developed and trained with 378 patients from a single Korean hospital, and the authors demonstrated external validation by testing it in 298 patients from three other institutions. Artery-specific AUC was 0.919 (95% CI, 0.893–0.942) with 92.7% sensitivity and 98.5% negative predictive value (NPV); patient-specific AUC was 0.871 (95% CI, 0.833–0.906) with 93.3% sensitivity and 96.6% NPV [14]. These results suggest safe deferral of invasive catheterization in patients with low-to-moderate pretest probabilities of ACS, while sending those with higher pretest probabilities for earlier angiography, consistent with the 2023 ESC guidelines for ACS [31]. Because it is derived from a single country, the model’s cross-geographic generalizability will be limited. Zhang et al. [15] identified culprit lesions using the entire diagnostic process to develop a multivariate model, which they applied to 491 patients admitted for acute myocardial infarction who subsequently underwent CCTA within 48 h across several sites. They combined CAD-RADS, high-risk plaque characteristics, and ΔFFR-CT into a single variable. The areas under the receiver operating characteristic curve (AUC) were 0.877 (95% CI, 0.847–0.906) and 0.853 (95% CI, 0.817–0.885), respectively. The addition of radiomics did not yield a statistically significant improvement over the model without radiomics, consistent with the authors’ conclusion that radiomics provided no information beyond anatomical and hemodynamic assessment.

### 5.2. AI-Derived Fractional Flow Reserve in NSTE-ACS

AI-derived fractional flow reserve in NSTE-ACS received its first prospective validation by Meier and colleagues [37]. Across four European centers, 168 high-risk NSTE-ACS patients were enrolled prospectively. After exclusions for heart rate (*n* = 4) and image quality (*n* = 13), 151 were included in the final analysis. Vessel-level discrimination favored FFR-CT decisively over anatomical CCTA alone: AUC 0.85 versus 0.64 (*p* < 0.01). Per-patient sensitivity reached 94% with an NPV of 85%, a configuration appropriate for a rule-out tool. The per-patient accuracy comparison did not reach significance (*p* = 0.58), but the vessel-level signal is where the cath-lab decision lives. Gwizdala and colleagues added a parallel test in 80 NSTE-ACS patients from the same Lausanne project, training a learned-fusion ML model on two orthogonal, multiplanar reconstructions per segment. The resulting AUC of 0.84 ± 0.06 matched that of FFR-CT (0.82 ± 0.08), with specificity 0.93 ± 0.05 and sensitivity 0.55 ± 0.14 across 514 segments at a culprit prevalence of 12.3% [38]. The high-specificity/low-sensitivity profile fits a confirmatory adjunct rather than a screening layer—culprits would be missed in low-pretest cases, but false positives are uncommon when the model triggers.

Two methodological qualifications are worth stating plainly. Image quality remains the gating constraint—10% of the Meier cohort was excluded for heart rate or image quality reasons, and exclusion rates rose to 44% of scans entering a low-quality subset in Glessgen’s opportunistic-aortic-CTA setting [37,39]. Whether the fluid-dynamic boundary conditions used in FFR-CT computation transfer cleanly to coronary lesions undergoing acute plaque rupture with intracoronary thrombus and microvascular dysfunction remains an open empirical question; the available evidence is consistent with degradation, particularly in cohorts enriched for acute occlusion. Photon-counting CT, now in clinical deployment, is expected to compress the low-quality fraction by handling calcified plaque without blooming, but prospective ACS data remain pending.

### 5.3. AI Plaque Phenotyping and Pericoronary Adipose Tissue

AI plaque phenotyping has developed on two levels. The first is that Ihdayhid et al. [40] validated the cross-modal anchor by comparing DeepPlaque 1.0 (HeartFlow, Inc., Mountain View, CA, USA) with IVUS in 67 non-culprit arteries from 33 STEMI patients who had undergone CCTA 2–40 days post-primary PCI. Correlations at the vessel level were 0.94; at the lumen volume, 0.97; at the total plaque volume, 0.92; at the non-calcified plaque, 0.91; at the calcified plaque, 0.87. This work demonstrates the feasibility of using artificial intelligence and computed tomography angiography compared to traditional invasive methods. The second is that Ferkh and coworkers [33] translated quantification into prognosis: In a group of 527 Emergency Department patients stratified by hs-cTnT level, the Autoplaque 3.0 (Cedars-Sinai Medical Center, Los Angeles, CA, USA) tool assessed the 315 CAD-RADS-positive scans (302 of 315 analyzable). When the total plaque burden in those with CAD-RADS-positive images exceeded 250 mm^3^, there was a statistically significant increase in the Hazard Ratio for Major Adverse Cardiovascular Events over a median follow-up period of 29 months (22 events) with an HR of 2.62 (95% CI, 1.13–6.07; *p* = 0.02). Although the multivariable model that included total plaque burden, age, hs-cTnT, and quantitative stenosis did not reach statistical significance (*p* = 0.18), this demonstrated that total plaque burden predicted major adverse cardiovascular events better than hs-cTnT in these patients. This study clearly shows that troponins reflect what has already occurred, while AI-assessed plaque burden will inform us about what could occur in the future. Beyond burden alone, Koo and colleagues showed in the EMERALD-II cohort that AI-enabled quantitative plaque and hemodynamic analysis added to standard CCTA assessment improved prediction of ACS culprit lesions (AUC 0.84 vs. 0.78; *p* < 0.001), with fractional flow reserve across the lesion, plaque burden, total and low-attenuation plaque volume, and percent myocardial blood flow as the strongest features [34].

Pericoronary adipose tissue (PCAT) has become evident as a CT marker of local vascular inflammation. Jing et al. [35] provided the first Tier 1 acute-diagnosis evidence: Among 620 patients (149 ACS scanned within 3 days of admission, 227 CCS, 244 no CAD), PCAT radiomics surrounding all three principal arteries identified ACS from CCS with an AUC of 0.86–0.94 and ACS from no-CAD with an AUC of 0.87–0.95, indicating that the Right Coronary Artery Fat Attenuation Index was the best differentiator. China’s National Medical Products Administration cleared the system (CoronaryDoc, Shukun Technology Co., Ltd., Beijing, China) as a Class III Device employing ResU-net + V-net segmentation; however, this was tested retrospectively at a single site, without STEMI/NSTEMI stratification or international regulatory approval, and thus prospective multicenter validation is required before widespread utilization [35]. For the practicing interventionist, there are three questions. Can AI-based CCTA safely delay invasive catheterization in suspected Non-ST-Elevation Acute Coronary Syndrome (NSTE-ACS)? Cautiously positive results have been reported by Kim and Meier [14,37]. Is the AI-determined culprit lesion viable until the time a wire crosses it? While prospective validation is encouraging [36] it remains unproven. Does AI plaque phenotype provide improved interval medical management? Consistent results have been reported by both Ihdayhid [40] and Ferkh [33] for plaque burden assessment, although the FAI question remains unanswered. CCTA is not appropriate for confirmed ST-segment elevation. Door-to-balloon time does not allow time for a scan, and the electrocardiogram continues to serve as the gatekeeper at the bedside during STEMI admissions. Rather, CCTA provides intermediate-level confidence when the electrocardiogram is nondiagnostic and troponin levels are indeterminate, setting the stage for anticipated subsequent trial evidence.

## 6. AI in Invasive Coronary Angiography and Coronary Physiology

The catheterization laboratory is the space where the operator and artificial intelligence (AI) confront a persistent, reproducible disagreement. Inter-observer agreement for visual estimation of the degree of stenosis has long been known to be poor, and even central core-lab adjudication does not eliminate this variability. In stable coronary artery disease (CAD), operators view lesions under elective conditions. However, when dealing with acute coronary syndromes (ACS), the treatment threshold for the intermediate non-culprit lesions seen in many STEMI patients falls within the visually ambiguous portion of the spectrum. Four types of AI have entered the cath lab. These include: anatomically-based AI (segmentation of the lumen, grading of stenosis); physiologically-based AI (FFR derived from angiography); workflow-based AI (fusion of roadmap, enhancements for stenting, reductions in contrast and radiation doses); and outcome prediction-based AI (bleeding post-procedure, AKI, MACE). The published evidence on these four types of AI, specifically addressing ACS, is inconsistent.

### 6.1. AI-Augmented Quantitative Coronary Angiography—The FLASH Trial

The first randomized study to assess AI-enhanced quantitative coronary angiography (AI-QCA) was the FLASH study. Kim et al. [12] randomly assigned 400 patients (395 in the primary endpoint analysis) who underwent PCI at 13 sites in South Korea during October 2022 through February 2024 to either receive an AI-QCA-assisted PCI using the MPXA-2000 system (Medipixel, Inc., Seoul, Republic of Korea; separate validation for AI-QCA vs. manual QCA performed in a multicenter cohort of 1076 angiographic images with 93% lesion detection sensitivity, Chae et al. [41]) or to receive OCT-guided PCI. The primary non-inferiority endpoint was the post-procedural minimum stent area (MSA), as determined from core-laboratory OCT data. AI-QCA was noninferior to OCT for MSA.

OCT is the gold standard for coronary artery imaging, not an alternative treatment strategy; non-inferiority of the minimum stent area (MSA) with AI-QCA versus the gold-standard measurement therefore supports AI-QCA as a substitute for that measurement, but does not support substituting AI-QCA for intracoronary imaging itself, whose appropriate comparator would be manual QCA or visual estimation. The larger difference in malapposition rates (13.6% with AI-QCA vs. 5.6% with OCT; *p* = 0.007) is thus the more important finding and suggests that AI-QCA should not replace intravascular imaging for assessing complex coronary lesions. FLASH enrolled predominantly stable or silent ischemia, with acute coronary syndrome (ACS) presentations a minority (unstable angina and acute myocardial infarction together about 41%, with no ACS-specific analysis), which limits its external validity in the ACS setting. Further limitations include exclusion of left main disease, chronic total occlusions, bypass grafts, and bifurcation lesions requiring two-stent techniques. These represent many of the same clinical contexts in which intracoronary imaging would provide the greatest value. Thrombi typical of ACS culprits can distort lumen contours and introduce bias into automated edge detection systems. Acutely constricted segments may have smaller-than-expected reference diameters. Therefore, we see utility for AI-QCA as a screening and quality control tool for non-complex lesions where access to OCT or IVUS is limited.

### 6.2. Angiography-Derived Fractional Flow Reserve—FAVOR III and ALL-RISE

Angiography-derived fractional flow reserve (FFR) has been studied in three clinical trials. Two produced conflicting results; the third further complicates a meta-analysis: FAVOR III China [3], FAVOR III Europe [4], and ALL-RISE [5]. These trials did not test a single technology: FAVOR III China used QFR computed with AngioPlus (Pulse Medical, Shanghai, China), FAVOR III Europe used QFR computed with QAngio XA 3D (Medis Medical Imaging Systems, Leiden, The Netherlands), whereas ALL-RISE used FFR-angio computed with the FFRangio System (CathWorks, Kfar Saba, Israel)—distinct algorithms from different vendors with different validation profiles—so the divergent results may partly reflect platform-specific differences rather than a limitation inherent to the angiography-derived FFR concept.

FAVOR III China [3] randomized 3825 patients with either stable coronary artery disease (CAD) or low-risk acute coronary syndromes (ACS) to PCI based on either angiography-derived quantitative flow ratio (QFR) or angiography alone. Case mix matters: 58.1% of patients had troponin-negative unstable angina, 25.8% had stable angina, 10.7% had asymptomatic ischemia, and 5.3% had a myocardial infarction 72 h to 30 days before enrollment. Excluded were those with acute myocardial infarction within 72 h of enrollment, severe heart failure, or severe chronic kidney disease (eGFR < 45 mL/min/1.73 m^2^). Compared with angiography-guided PCI, one-year rates of major adverse cardiac events (MACE) were significantly lower with QFR guidance (5.8% vs. 8.8%; hazard ratio, 0.65; 95% CI, 0.51–0.83; *p* = 0.0004), primarily due to fewer myocardial infarctions and ischemia-driven revascularizations. The difference was sustained over two years (hazard ratio 0.66, 95% CI 0.53–0.81) [42]. QFR altered the intended plan of care for about 23.3% of participants, with approximately 19.6% having at least one vessel deferred from intervention, resulting in shorter fluoroscopy time (14.1 ± 8.0 min vs. 14.9 ± 7.4 min; *p* = 0.0013). FAVOR III Europe reversed this. Andersen et al. [4] enrolled 2000 patients with either chronic coronary syndrome or stabilized ACS who underwent angiography and compared QFR with pressure-wire measurement. The primary endpoint (death, MI, or unplanned revascularization) occurred in 6.7% of QFR patients versus 4.2% of FFR patients (HR, 1.63; 95% CI, 1.11–2.41); the event-proportion difference was 2.5% (90% CI, 0.9–4.2%), with the upper bound exceeding the prespecified 3.4-percentage-point non-inferiority margin. The trial failed. Patients treated with QFR had 21% higher rates of study-lesion revascularization and 27% higher rates of stent implantation (823 vs. 650). The median QFR (0.81) was lower than the median FFR (0.84), and QFR classified more left circumflex lesions as functionally significant than FFR (37.1% vs. 15.4%). A post hoc deferral analysis demonstrated significantly higher rates of major adverse cardiac events (adjusted hazard ratio 2.07, 95% CI 1.07–4.03) and of target-vessel failure restricted to study vessels (adjusted HR 2.27, 95% CI 1.00–5.16) among patients deferred using QFR than among FFR-deferred patients [43]; both false positives and false negatives therefore appear to have occurred with QFR. A repeatability analysis [44] found a high degree of measurement variability, which increased with suboptimal angiographic and in-procedure assessment quality.

ALL-RISE reads as the platform-specificity counterpoint. In their randomized trial, Fearon et al. [5] randomly assigned 1930 patients across 59 centers worldwide to either FFR-angio (using CathWorks) or pressure-wire FFR for intermediate coronary lesions. Only about 9 percent of these patients had elevated cardiac biomarkers before undergoing catheterization. Culprit vessels identified during recent STEMI and UA/NSTEMI events were also excluded, which means this patient population isn’t representative of acute decision-making regarding the culprit vessel. The pooled rate of death, myocardial infarction (MI), or unplanned revascularization at one year post-procedure was similar with either technology (FFR-angio: 6.9%, Pressure Wire: 7.1%), with HR = 0.98 (95% CI, 0.70–1.39) and *p* < 0.001 for non-inferiority. Additionally, procedures utilizing FFR-angio took significantly less time than those involving a pressure-wire (39 min vs. 42 min), produced less radiation exposure (fluoro-time = 9 min vs. 12 min), and resulted in a higher frequency of PCI utilization (44.3% vs. 35.4% of study lesions; OR = 1.45 (95% CI, 1.24–1.70)—again, representing the same angiography-versus-wire bias observed in FAVOR III Europe, yet did not produce a negative clinical outcome signal. Therefore, All-RISE serves as a supportive data source for wire-free physiology, albeit strictly limited to stable, non-culprit, intermediate lesions. A randomized trial comparing angiography-derived FFR (QFR) with angiography-guided revascularization of non-culprit lesions in multivessel STEMI has completed enrollment (*n* = 1823); results are awaited (AIR-STEMI; NCT05818475) [45].

### 6.3. Workflow AI and Post-PCI Outcome Prediction

Workflow AI is the cath-lab category most operators already use without naming it. The Dynamic Coronary Roadmap (Philips Healthcare, Best, The Netherlands) uses live fluoroscopy and adds a vessel skeleton to correct for both respiratory and cardiac motion. In their randomized trial, DCR4Contrast, Hennessey et al. [46] randomly assigned 356 patients undergoing PCI to receive either DCR-assisted fluoroscopy or standard fluoroscopy. There was a significant decrease in iodinated contrast, from 90.8 ± 55.4 mL to 64.6 ± 44.4 mL (*p* < 0.001), approximately a 29% reduction, with fewer cineangiographic runs (8.7 vs. 11.7; *p* < 0.001), and the operators rated procedural success equally. The subanalysis by Quast et al. [47] indicated that, regardless of operator experience, roadmapping reduces contrast use. Temporal-averaging stent-enhancement software, StentBoost (Philips Healthcare, Best, The Netherlands) and ClearStent (Siemens Healthineers, Forchheim, Germany), although not considered ‘true AI,’ also fits into the operator’s workflow. A 2025 systematic review of 12 articles reported that StentBoost improves detection of stent under-expansion and deployment compared with standard angiography and shows a high correlation with OCT and IVUS [48]. Although they may not be truly AI, other technologies such as contrast-reducing hardware (DyeVert, Osprey Medical, Minnetonka, MN, USA) and adaptive-dose optimization software (Allura Clarity, Philips Healthcare, Best, The Netherlands) fit into this category as well; in a multicenter observational study [49], the average contrast reduction was 40.1 ± 8.8% among the 114 patients with an estimated glomerular filtration rate of 20–60 mL/min/1.73 m^2^. Outcome-prediction models using machine learning have begun to replace traditional post-PCI risk scores, though the gains are relatively small. Mortazavi et al. [13] evaluated 3,316,465 PCI procedures from the NCDR CathPCI registry. Their analysis demonstrated that an XGBoost model achieved a c-statistic of 0.82 for predicting bleeding, compared with 0.78 for the complete NCDR model, correctly identifying an additional 3.7% of high-risk bleeding cases and reducing the false-discovery rate from 78.7% to 73.4%. The PRAISE score (developed by D’Ascenzo et al. [50]), using data from 19,826 ACS patients across BleeMACS and RENAMI, achieved an external-validation AUC of 0.86 for one-year major bleeding, a rare example in which external performance exceeded internal performance. For post-PCI AKI, Kuno et al. [10] used a LightGBM model with an AUC of 0.772 based on only seven SHAP-selected variables (age, eGFR, pre-procedure hemoglobin, STEMI status, NSTEMI/UA, HF symptoms, and cardiogenic shock). Sun et al. [51] validated the trend in 1495 AMI patients, with a random forest AUC of 0.82, compared with 0.69 for logistic regression and 0.62 for ACEF. These differences are small, but they matter where decisions on contrast volume, pre-hydration, and discharge timing hinge on probabilities that traditional scores cannot resolve. Table 2 consolidates the AI evidence base across biomarker, CCTA, invasive angiography, and intracoronary imaging applications in ACS.

## 7. AI in Intracoronary Imaging—OCT and IVUS

Intracoronary imaging provides the “substrate” information that an angiogram’s silhouette cannot provide (plaque erosion vs. rupture, thin-cap fibro-atheroma, residual thrombi, stent malposition, and edge dissections). The clinical and prognostic relevance of differentiating among these entities in patients with acute coronary syndromes is established—25–40% of ACS are caused by plaque erosion [53], which is associated with platelet-rich thrombi and often occurs at a younger age than those who experience plaque rupture, with some studies reporting that this morphology was more commonly found in women in previous reports; plaque erosion may be an indication of a conservative antithrombotic regimen rather than stent placement [54]; thin-cap fibro-atheromas represent the substrate for plaque rupture; stent under-expansion and mal-apposition contribute to both stent thrombosis and restenosis. Artificial intelligence (AI) applied to Optical Coherence Tomography (OCT) and Intravascular Ultrasound (IVUS) has two clear uses. One is to accelerate and standardize procedure-related measurements, since human readers perform these tasks slowly and inconsistently. The second is diagnostic and prognostic, in which pattern recognition identifies what the eye misses.

### 7.1. Automated Lumen and External Elastic Lamina Segmentation

At the procedural level, automated segmentation of the lumen and external elastic lamina (EEL) removes the bottleneck of manual annotation. Results are impressive. Of the several architectures tested, DeepLabv3+ achieved the best Dice similarity coefficients, 0.927 and 0.944 for lumen and EEL contours, respectively, across 6516 IVUS frames from 175 pullbacks collected at multiple centers [52]. Another study used a high-frequency IVUS (60 MHz) neural network developed on 8076 images from 234 patients and, in an independent test set, reported a 92.4% agreement rate for balloon sizing against the expert reference when both vessel and lumen diameters were used (70.6% by vessel diameter alone), with a Pearson correlation of 0.99 for lumen and vessel area versus the hand-drawn reference in the training set [55]. As stated earlier, the pre-procedure determination of stent under-expansion follows the same logic described above. For example, the CNN-plus-XGBoost model developed by Min [9] demonstrated a 94% accuracy and an area under the curve (AUC) value of 0.94 when predicting post-procedure areas <5.5 mm^2^ based on pre-procedural IVUS imaging combined with procedural parameters, thus allowing for the potential for prior upsize, increased inflation pressure, or post-dilatation during subsequent procedures. Although the substrates these AI algorithms use to make predictions (i.e., lumen contour, vessel wall, calcification arc) may be similar in both stable and acute coronary syndromes (ACS), none of the studies referenced used ACS-defined cohorts; therefore, outcome-level transferability of IVUS-AI to ACS has not been demonstrated.

### 7.2. Plaque Characterization and Risk Stratification—OCT-AI

At the diagnostic and prognostic level, OCT-AI is supported by ACS-defined evidence. Using the transformer architecture developed by Park et al. [11], and training their models on 237,021 OCT images obtained from 581 ACS patients and then validating them on 65,394 images from 292 additional ACS patients, they were able to achieve high accuracy using a single OCT frame for detecting plaque erosion with an Area Under Curve (AUC) of 0.94 compared to 0.85 when they used a Convolutional Neural Network (CNN) as the comparative. They also demonstrated high sensitivity (0.927) and specificity (0.885), allowing clinicians to safely use a conservative antithrombotic strategy without stenting in selected ACS patients with intact infarct-related arteries. In addition to testing the accuracy of their OCT-AI algorithms for detecting TCFA on a per-frame basis, Volleberg et al. [8] conducted a secondary analysis of the prospective PECTUS-obs study, applying OCT-AI to detect TCFA across the complete imaged segment in MI patients. Of the 438 patients who underwent OCT imaging, 414 were included in the primary analyses after exclusions. Their results show that the OCT-AI algorithm detected TCFAs in 34.5% of their patient population, whereas expert core laboratory readers detected TCFAs in only 30.0% of the same patients (kappa = 0.40). When limited to the target lesion, the AI-detected TCFA predicted 2-year MACE events with a hazard ratio of 1.99 (95% CI 1.02–3.90; *p*-value = 0.04), whereas the expert-read TCFA at the target lesion did not predict 2-year MACE events (hazard ratio = 1.67, 95% CI = 0.84–3.30; *p*-value = 0.14). However, when they analyzed the data across the entire imaged segment, they found a significant increase in the prediction of 2-year MACE among patients whose vessels contained any TCFAs identified by the AI (*p* < 0.001), with a hazard ratio of 5.50 (95% CI 1.94–15.62). Additionally, they determined that if no TCFA was detected anywhere in the pullback, the risk of a major adverse cardiac event during follow-up was very low (negative predictive value = 97.6%, 95% CI 94.0–99.3%).

Chu et al. [56] reported on how a convolutional neural network and an encoder-decoder architecture were used to enhance OCT assessments of the morphology and function of atherosclerotic plaques. The researchers designed a pseudo-3D input layer inside the OctPlus software (Pulse Medical Imaging Technology in Shanghai, China). This model locates the internal elastic lamina and lumen boundaries, classifies the intervening tissue as lipidic, calcific, or fibrous, and identifies macrophages, cholesterol crystals, and microvessels as indicators of complex and inflamed plaques. Altogether, 509 intravascular OCT pullbacks from 391 patients with stable lesions were used to train the models. For each tissue feature, the Dice coefficient was high against manually segmented core-laboratory data for structural components (fibrous: 0.906, calcific: 0.848, lipidic: 0.772) but poor for inflammatory markers (macrophages: 0.489, cholesterol crystals: 0.525). The correlation with manual plaque-burden measurement was strong (R^2^ = 0.98). Per-component accuracy for fibrous, lipidic, and calcific plaque was 97.6%, 90.5%, and 88.5%, respectively, when tested against three international core laboratories, but only 48.1% of macrophages were correctly identified. Hong et al. [57] evaluated two OCT-based metrics, the automated lipid-to-cap ratio (LCR) and optical flow ratio (OFR), derived from a single OCT image, for their relationship to major adverse cardiac events (MACE) at 2 years after acute coronary syndrome among 604 consecutive patients retrospectively recruited from a single hospital in China. Kaplan-Meier and Cox regression analyses showed that the 96 patients meeting both criteria (LCR > 0.33 and OFR ≤ 0.84) experienced 23 events (24.0%) and had a hazard ratio of 42.73 (95% CI 12.80–142.60; *p* < 0.001) versus the other 508 patients in whom at least one metric was normal (3 events; 0.6%). Each metric individually also predicted MACE (HR 19.13, 95% CI 4.49–81.55 for LCR > 0.33; HR 11.52, 95% CI 2.70–49.12 for OFR ≤ 0.84). The extremely large combined hazard ratio should be regarded as hypothesis-generating, pending prospective multicenter testing.

With only 23 events in the high-risk group in a single-center retrospective design, a hazard ratio of this magnitude is almost certainly inflated by overfitting, sparse-event bias, and unmeasured confounding and should not be interpreted as a stable effect size. Both LCR (AUC 0.826; 95% CI 0.793–0.855) and OFR (AUC 0.838; 95% CI 0.806–0.866) outperformed minimal lumen area (AUC 0.618; 95% CI 0.578–0.657) in predicting MACE, and together they defined risk groups more clearly than either alone. Collectively, these studies suggest that structural plaque features can be measured using artificial intelligence (AI), whereas cellular inflammatory characteristics, such as macrophage density, need further development. The prospective, multi-institutional PREDICT-AI study [58] will test whether AI-assisted OCT plaque assessment of all three coronary arteries predicts outcomes after ACS, scoring six features (lipid arc, fibrous-cap thickness, macrophage presence, cholesterol-crystal presence, minimum lumen area, calcified-nodule presence) against a primary MACE endpoint (cardiovascular death, myocardial infarction, urgent revascularization, and target-vessel revascularization); it is recruiting, with no results yet published.

### 7.3. Structural Caveats and Practical Synthesis

There are three structural limitations in the literature on AI for OCT/IVUS in ACS that are common across all studies. First, there is a notable lack of research. A scoping review identified a few studies that specifically address the use of AI to analyze 60 MHz high-definition IVUS images [59]. The IVUS-ACS randomized clinical trial demonstrated a 45% reduction in target vessel failure with IVUS-guided PCI compared with angiography-guided PCI in patients presenting with acute coronary syndromes (HR 0.55, 95% CI 0.41–0.74; *p* = 0.0001) [60]. Second, geographic location and race may be barriers to the inclusion of South Asian, Sub-Saharan African, Latin American, and Middle Eastern populations in both OCT and IVUS AI trials. The PECTUS-AI cohort comprises subjects who were predominantly white and represents a secondary dataset of PECTUS-Obs [8]; Park’s Transformer was developed and validated using data from an international, multicenter patient population of East Asian, European, and North American sites [11]; Min et al.’s IVUS-AI predictor of under-expanding stents was developed and tested in Korean patients with CAD [9]; Garg et al. found the reproducibility of AI-based OCT tissue classification was good in 74 STEMI patients; however, they studied only a single-vendor OCT image-analysis software system [61]. Third, these AI pipelines are vendor-specific and heterogeneous; some have full development pipelines built on proprietary OCT platforms [8,11,62], whereas others are analysis software packages (e.g., OctPlus, Pulse Medical) [56,61]; applied primarily to OCT images acquired with Abbott systems. These pipelines have undergone little formal evaluation on 60 MHz high-definition IVUS (HD-IVUS) or on newer commercially available AI overlays such as Ultreon 3.0 (Abbott, Santa Clara, CA, USA; FDA-cleared and CE-marked April 2026) [62] and AVVIGO+ (Boston Scientific, Marlborough, MA, USA), leaving the impact of this limitation on ACS-related detection unknown.

## 8. Multimodal Integration and Cross-Modality Risk Stratification

AI in the ACS care setting serves as a secondary, parallel advisory layer to the traditional cascade model rather than as a component of that cascade. Its first application is at the ambulance level, where AI-ECG can identify an acute myocardial infarction caused by occlusive coronary artery disease before the patient reaches the hospital entrance. The sensitivity of AI-based ECG analysis exceeds that of STEMI-specific electrocardiographic criteria. On arrival in the Emergency Department, machine-learning algorithms interpret high-sensitivity troponin levels and produce continuous numerical outputs that allow the physician to assign each patient a low, intermediate, or high probability of having had a myocardial infarction [16,27]. Patients with non-ST-elevation acute coronary syndromes (NSTE-ACS) are evaluated for invasive catheterization using AI-based CCTA; because of its high negative predictive value, this approach avoids unnecessary procedures [37,38]. During catheterization, AI-based QCA provides standardized measurements of stenoses [14]; angiography-derived fractional flow reserve (FFR) provides physiological assessment when guidewire placement is impractical [5]; and intracoronary imaging AI provides morphological information that may guide therapeutic intervention before subsequent events [8,11].

The pathway integration breaks down into three operational questions: Where do the modalities reinforce each other? Where do they conflict? What specific functions of AI are difficult for human readers to replicate? The strongest example of reinforcement occurs early in the process—an abnormal AI-ECG OMI score (Al-Zaiti et al. [1]) and an abnormal hs-cTnI delta over 3 h [27], together produce a very strong probability call for catheterization without additional testing. Reinforcement also exists later, after the wire has reached the heart: AI-CCTA plaque burden [40] and OCT-derived plaque features [58] point in the same direction toward intensified medical therapy. Conflict arises in two settings. First, during the initial hours of OMI, AI-ECG can be the sole indicator of OMI while troponin remains within its reference range—a costly diagnostic delay that the OMI paradigm of Meyers et al. [2] was developed to prevent. Second, AI-based physiologic measures (specifically, QFR) have been shown to overestimate lesion significance in the circumflex/obtuse marginal territory: in FAVOR III Europe, Andersen et al. [4] found that 37.1% of circumflex/obtuse-marginal stenoses were designated functionally significant by QFR versus 15.4% by FFR, and QFR failed to demonstrate non-inferiority to the pressure wire (HR 1.63, 95% CI 1.11–2.41)—a single-trial finding in a relatively stable, intermediate-stenosis population that the authors noted had not been previously described. The third question has the cleanest answer. The Al-Zaiti et al. [1] model detected subtle, sub-STEMI ischemic patterns that experienced readers routinely miss, raising sensitivity by roughly 28 percentage points over reference standards and reclassifying one in three chest-pain patients when paired with clinician judgment—pattern recognition in a high-dimensional ECG space that few human readers reliably reproduce.

Beyond diagnosis, machine learning for post-event risk stratification has begun to refine traditional scores. Toprak et al. reported an analysis of the ARTEMIS-POC cohort that identified a safety signal: at the predefined rule-out threshold of less than 0.5%, as described in Section 4 [28], the proportion of index MI cases missed under the ML-driven rule-out criteria was 0.05% (95% CI, 0.04–0.42), and the 30-day cumulative incidence of the composite of myocardial infarction or cardiovascular death among patients ruled out by the ML criteria was 0.07% (95% CI, 0.06–0.59). Thus, the application of ML-based rule-out to preserve short-term patient safety exceeds what is achievable through improved diagnostic accuracy alone. Machine learning has also been applied to predict ACS complications rather than the index diagnosis: the STOP SHOCK score predicts a patient’s risk of developing cardiogenic shock in hospital based on variables that can be obtained during initial medical contact; using data from 5123 consecutive ACS patients, it had a c-statistic of 0.844 (95% CI, 0.841–0.847) on external validation [63]. This “implementation gap” is addressed by Section 9 and Section 10: (1) limited external validation; and (2) a measured decline in performance under independent testing across the ICU AI literature, where only 84/572 (14.7%) studies were externally validated and AUROC declined by 0.037 (95% CI, 0.027–0.052) on independent datasets [64]. These limitations define a practical upper limit on what is achievable with such post-event ML applications. As an example of precision therapy, Lee et al. [65] used X-learner machine learning to develop a personalized predictive score for the duration of dual antiplatelet therapy based on pooled data from 6568 patients enrolled in three clinical trials. Validation occurred within the TICO cohort, which included 3056 ACS patients. The model defined a subgroup predicted to derive greater benefit, in which a 3-month versus standard 12-month DAPT regimen yielded a lower absolute risk of major bleeding (absolute risk difference 1.26 percentage points; 95% CI, 0.15–2.36) with no significant difference in major adverse cardiac and cerebrovascular events (ARD 0.63 percentage points; 95% CI, −0.34 to 1.61). The model categorized 2582/3056 (84.5%) of subjects into this higher-benefit subgroup. While this is not an evidence base for routine deployment of the model—the original trials were not designed to validate it—it provides a methodological proof of concept that AI can identify treatment-effect heterogeneity in pooled clinical-trial data that traditional subgroup analysis cannot resolve.

The architecture that could lead to an integrated approach is likely a multimodal training model that uses all data streams handled independently today; however, we have a body of engineering experience from prior work. We know how to combine large-scale self-supervised pre-training (such as Cho et al. [21] for AI-ECG), end-to-end deep learning models (e.g., Herman et al. [7,20]), and interpretable feature engineering (e.g., Al-Zaiti et al. [1]) to produce a single system. There are federated learning frameworks available today to support the development and deployment of models that can be trained across multiple heterogeneous institutional cohorts without collecting patient-level data—thereby providing a technological solution to the geographic representativeness issue described in Section 9. This technology is not used in clinical practice today, nor do we understand what will constitute the regulatory path forward for ongoing-learning multimodal models based upon either the EU AI Act or the FDA SaMD framework.

## 9. Implementation, Regulatory Status, Equity, and Generalizability

The regulatory environment for AI in cardiovascular medicine varies significantly by region and by stage on the continuum. For example, Powerful Medical’s PMcardio platform, which includes eOMI v1 [7] and aOMI v1/Queen of Hearts [20] and holds the CE mark, is, to our knowledge, the only CE-marked ECG-AI device for detecting acute-occlusion MI as of submission. The same device received FDA Breakthrough Device designation in March 2025; however, it has not been approved for commercial distribution in the US. AI-assisted CCTA is further along in the approval process: HeartFlow’s FFR-CT has had FDA clearance since 2014 via the De Novo pathway, and there are now formal recommendations to standardize the quantification of AI-derived plaque burden to enable individualized treatment decisions for patients with atherosclerotic disease [66]. AI-based quantitative coronary angiography has entered RCTs; FLASH used the Medipixel MPXA-2000 in approximately 400 patients [12], though its use remains geographically limited. Several companies have developed angiography-derived FFR algorithms (QFR and FFR-angio); however, these were primarily validated in populations with stable CAD or intermediate lesions rather than ACS: QFR-guided PCI produced better outcomes than angiography-guided PCI in FAVOR III China [3], whereas QFR failed to demonstrate non-inferiority to wire-based measurement in FAVOR III Europe [4], and FFR-angio was non-inferior to wire-based measurement in ALL-RISE, a trial that enrolled mainly stable-CAD patients without STEMI [5]. AI-based intravascular imaging has also entered clinical use, but prospective data on its ACS-specific benefit remain lacking. However, prospective data on ACS-specific benefit remains lacking.

Three implementation constraints recur. First, the training dataset. The datasets used by Al-Zaiti, Herman, and Cho came from North America, Europe, and East Asia but included too few South Asian, sub-Saharan African, and Latin American subjects [1,11,12,13]; since algorithmic performance depends on both baseline electrical pattern and the demographic distribution of training data, validation in underrepresented populations is the most important gap in the literature. Second, calibration to sex. Wenzl and coworkers [29] showed that the legacy GRACE 2.0 score had lower predictive capability in women than in men, with an AUC difference of about 0.04. AI-ECG models trained on predominantly male data may share this limitation, yet none of the published validation cohorts have been evaluated by sex with sufficient granularity. The same group redeveloped the sex-specific GRACE 3.0 score against GRACE 2.0. Third, the performance-explainability trade-off. Engineered-feature models, such as Al-Zaiti’s ECG-SMART [1], enable SHAP-level inspection of why an ECG was flagged. In contrast, end-to-end deep-learning models such as Herman’s eOMI [11], a deep convolutional neural network, may achieve marginally higher discrimination but remain essentially black boxes, with no head-to-head comparison against engineered-feature models.

The European Union’s AI Act (Regulation (EU) 2024/1689) classifies medical AI as high-risk; from 2026 through 2027, each new high-risk medical AI application will require pre-market conformity assessment, post-market surveillance, and documented lifecycle risk management. The U.S. approach differs. The FDA’s software-as-a-medical-device framework allows ‘locked’ algorithms whose performance is fixed after clearance, while models that keep learning follow a predetermined change-control plan (PCCP) set out in finalized guidance (Docket No. FDA-2022-D-2628). There are no standing programs to support this route to certification: the FDA ended its software precertification pilot in 2022, deciding that an operational version would require new legislation. A second pilot, Technology-Enabled Meaningful Patient Outcomes (TEMPO), began in December 2025 and provides risk-based enforcement discretion under the CMS ACCESS model for technology-enabled digital health devices used by clinicians for outpatient management of chronic diseases [67]. It is neither a precertification program nor applicable to acute AI-ECG triage in ACS. We know of no ACS-specific AI device that has been admitted into either a precertification or TEMPO pathway. Both frameworks face the same challenges: data, population, and practice drift during deployment. An AUC of 0.94 in 2023 does not guarantee the same in 2027, because clinical practice evolves, case mix changes, and even the assay biomarker may change, which is why local validation is now seen as part of operationalization rather than a one-off market-entry barrier. The evidence for degradation is concrete: a systematic review of machine-learning risk scores found a mean AUROC decline of −0.037 (95% CI, −0.052 to −0.027) on external datasets, with drops greater than 0.05 in 49.5% of externally validated models [64]. These models addressed ICU deterioration rather than ACS, but dataset shift does not respect diagnostic boundaries. Each constraint also carries an equity cost: hardware alone, GPU inference, PACS and EMR integration, and high-bandwidth connectivity skew where these models are deployed. Edge computing, federated learning across sites without moving patient data, and cloud-based inference for remote locations have been proposed, but none have been tested at scale. The recommendation is operational, not aspirational: funders should require diverse populations in validation studies, regulators should weigh population representativeness in approval decisions, and professional societies should develop guidelines for deploying AI across resource settings.

The discriminative ECG-AI models we have discussed demonstrate several characteristic failure modes beyond those already mentioned. Unlike generative models, which can produce fabricated information (hallucinations), discriminative models instead output a confident but incorrect classification when given data they did not see during training—such as paced rhythms, heavily artifacted recordings, misplaced leads or electrodes, or morphologies not contained in the training set. Because these models provide no measure of confidence beyond a single probability value, many of these failures are “silent” and become apparent only as a gradual decline in discrimination as the model processes patients from different demographics or data from different instrumentation. This increases the potential for automation bias when a clinician accepts a high-confidence prediction without independent verification. These failure modes argue for at least three safeguards in the development and deployment of future ECG-AI systems: (1) mandatory clinician review of model predictions before any action is taken; (2) models that express uncertainty in addition to a single probability, so clinicians can judge how reliable a prediction is; and (3) real-world performance monitoring after clinical deployment.

Table 3 summarizes the practical considerations for deploying AI in ACS care, spanning regulatory status, external validation, algorithmic equity, and cost.

## 10. Limitations of the Evidence Base

Five limitations of the available evidence base deserve explicit statement. First, the great majority of cited studies are diagnostic-accuracy reports rather than outcome trials. No randomized controlled trial has yet demonstrated that AI-ECG, AI-CCTA, AI-OCT/IVUS phenotyping, or ML risk scoring specifically reduces mortality in ACS compared with standard of care; the available trials report procedural-quality endpoints—minimum stent area in FLASH [12]— or MACE composites in which the signal is driven by ischemia-driven revascularization and myocardial infarction rather than mortality [3]. The DIFOCCULT-3 trial of an OMI/NOMI paradigm with AI-supported ECG analysis is enrolling but has not yet been reported [19]; AIR-STEMI, evaluating QFR in STEMI, has completed enrollment but has not yet been reported [45]. The translation of diagnostic accuracy into outcomes is the field’s most consequential pending question.

Second, convenience-cohort selection biases dominate the published validation studies. Cohorts assembled retrospectively from registries enriched for the index event over-represent the disease and inflate sensitivity estimates relative to a true population-based denominator. Where prospective enrollment has occurred [5,8,20], the disease spectrum is more representative, but the cohorts remain biased toward high-volume tertiary centers with research infrastructure.

Third, external validation rates remain low. The systematic review of AI-based scoring systems in the ICU by Rockenschaub [64] and colleagues reported that 14.7–23.9% of models had any external validation and a mean AUC decline of 0.037 on independent testing. Within the AI-ECG, AI-CCTA, AI-angiography, and AI-OCT domains reviewed here, the pattern is similar: only a minority of derivation studies have been prospectively validated in geographically distinct cohorts, and direct head-to-head architecture comparisons on shared external datasets remain absent. The Rockenschaub analysis is restricted to ICU AI scoring systems rather than cardiovascular AI per se, but the underlying mechanism—population, practice, and assay drift between derivation and deployment cohorts—applies directly to AI-ECG, AI-CCTA, and AI-OCT pipelines, and sets a realistic ceiling for any clinical-utility claim made on the basis of internal-validation metrics alone.

Fourth, spectrum bias affects models tested on STEMI-enriched cohorts. A model trained or tested on a cohort in which 21.6% of patients have OMI [7] operates in a fundamentally different prevalence space than the same model deployed in an emergency department, where 5% of chest-pain patients have OMI. PPV and NPV are prevalence-dependent; AUC is not, but the clinical interpretation of a fixed operating point changes substantially across prevalence settings. The Cho [21] external validation, with 0.42% AMI prevalence, illustrates this: PPV falls to 0.129, not because the model is poor, but because the prior probability is low.

Fifth, head-to-head comparisons between AI architectures are essentially absent. There is no published prospective trial comparing Al-Zaiti’s ECG-SMART [1], Herman’s eOMI [7,20], and Cho’s DeepECG-AMI [21] on a shared dataset; the available comparisons are all against human readers or against STEMI criteria, which are different reference benchmarks. The field has implicitly assumed that more recent architectures are better; the empirical evidence does not yet support that assumption with sufficient granularity. Pediatric, post-CABG, and chronic-coronary primary-screening populations have been systematically excluded across the diagnostic-accuracy literature.

## 11. Future Directions

The research priorities below follow from the evidence limitations and the regulatory and equity issues in Section 9 and are organized on a three-tier timeline. Within the first two years, three items are high-priority. First, completing and reporting the remaining ACS-specific outcomes trials. DIFOCCULT-3 [19] compares the OMI/NOMI paradigm, which integrates clinical gestalt, AI-supported ECG analysis, biomarkers, and imaging, with the STEMI/NSTEMI approach, with an estimated 6000 patients across 18 centers in Turkey; it is among the first prospective controlled trials to test whether this AI-supported paradigm improves timeliness and outcomes in suspected ACS. AIR-STEMI [45] will compare QFR-guided revascularization with conventional angiography-guided treatment in STEMI patients with multivessel disease. Both trials aim to translate diagnostic accuracy into clinical outcomes, a focus largely overlooked elsewhere. ALL-RISE’s finding that FFR-angio was non-inferior to the pressure wire can be cited only with explicit limiting language: 90.6% of the cohort had no elevated cardiac biomarkers, STEMI culprit vessels were excluded entirely, and NSTE-ACS culprit vessels were excluded by protocol [5]; the result applies to non-culprit, stabilized, intermediate-lesion decisions, not to the immediate culprit decision.

Second, mandatory reporting standards. Regulators and journal editors should require TRIPOD-AI reporting for diagnostic modeling studies and CONSORT-AI for interventional studies, with calibration metrics, decision-curve analyses, and fairness evaluations published routinely. Third, external validation should precede any claim of clinical utility, ideally through decentralized development across diverse geographies and ethnic groups beyond the training population.

Within two to five years, federated learning should be the top infrastructure priority: cooperative model training across providers assembles representative datasets without centralizing data, though it will require unified funding. Second, health-economic analysis will need to become routine as payers demand evidence of reduced downstream costs; few formal cost-effectiveness analyses exist for AI-QCA or AI-ECG, and the evidence for AI-CCTA, though more developed for FFR-CT, remains limited, thereby constraining reimbursement. Third, frameworks for continuously learning models are needed; neither the FDA’s predetermined change-control plan (PCCP) nor the EU AI Act provides a complete pathway for fully autonomous, ‘unlocked’ models. Beyond five years, the field may move toward multimodal foundation models trained jointly on ECG waveforms, troponin trajectories, CCTA volumes, IVUS frames, and EHR covariates. Single-modality proofs of concept already exist, such as multi-view machine learning for myocardial infarction from CCTA [38]. At the same time, a recent scoping review found only a limited number of machine-learning tools for intracoronary plaque imaging [59]. No multimodal system that integrates all patient-specific data into one risk score and a recommended action yet exists in clinical practice. The concept is in place, but large-scale multimodal training, handling of missing modalities, and prospective outcome evaluation must be resolved first. The human–AI workflow matters too: with no consensus on how to divide cognitive load, regulatory and ethical frameworks point to an advisory role in which the algorithm assists rather than replaces the clinician. Models should also alert operators when their performance degrades, since deployed accuracy cannot be assumed stable; longitudinal monitoring determines when recertification or withdrawal is required.

## 12. Conclusions

The current body of evidence establishes three general findings. First, as Herman and colleagues [7] demonstrated, AI-ECG algorithms can identify occlusive myocardial infarction (OMI) more accurately than the conventional STEMI criteria. Specifically, the first iteration of the eOMI algorithm, a deep neural network developed by them, detected OMI with 80.6% sensitivity, compared with 32.5% sensitivity for the conventional STEMI criteria. Subsequently, the same group [20] determined that use of the Queen of Hearts model significantly enhanced sensitivity when applied to patients being evaluated for OMI (increasing sensitivity by 21 percentage points [from 71.0% with standard triage to 92.0%]) and reduced false-positive cath-lab activations by 33.9 percentage points (from 41.8% to 7.9%). Al-Zaiti and colleagues [1] described an ECG-SMART model designed to evaluate the likelihood of an OMI developing among patients with symptoms of cardiac disease who have had an ECG. Results from their study indicated that the model accurately predicted whether an OMI would develop (AUROC = 0.87), compared with 0.80 for practicing cardiologists and 0.75 for a commercially available FDA-cleared device. Across these studies, sensitivity gains ranged from 21 to 48 percentage points. When high-sensitivity troponin analysis was combined with machine learning, it was possible to roughly double the number of patients who could be safely ruled out for myocardial infarction at ED presentation while maintaining a negative predictive value (NPV) greater than 99.5% [16,27,28]. One caveat constrains deployment: these troponin scores are assay-specific, so a model trained on one manufacturer’s high-sensitivity troponin assay cannot be assumed to generalize to another manufacturer’s assay without recalibration and revalidation. Although the results were similar across all cohorts examined, most of the established models are based on predominantly European, North American, and Australian populations. Therefore, whether they perform comparably across South Asian, sub-Saharan African, or East Asian cohorts (Section 9) remains unproven.

Second, several reports now demonstrate the utility of AI-assisted CCTA as a valuable diagnostic tool for ruling out suspected non-ST-elevation acute coronary syndrome and for guiding medical management between hospital admissions [14,37,38]. Machine learning has also improved manual assessment of intracoronary plaque morphology through AI-assisted diagnosis of plaque erosion [11] and identification of thin-cap fibroatheromas associated with adverse clinical outcomes [8]. Finally, current evidence does not permit a general conclusion about angiography-derived FFR, owing primarily to substantial between-platform variability in performance: FFR-angio was non-inferior to the pressure wire in ALL-RISE [5], whereas QFR did not meet its primary endpoint in FAVOR III Europe [4]. A great deal remains unknown. No completed randomized trial has shown that incorporating artificial intelligence into the evaluation and treatment of patients with acute coronary syndrome reduces mortality, recurrent MI, or ischemia-driven revascularization. Only a small fraction of potential AI applications have been externally validated, and this lack of external validation across settings is perhaps the biggest gap remaining in the field.

## Figures and Tables

**Table 1:** Summary of AI-ECG studies for OMI/STEMI/AMI detection.

Study	Endpoint	*n* (Test)	AUC (95% CI)	Sens/Spec	Comparator	Key Finding
Al-Zaiti 2023 [1]	OMI	3287 (external)	0.87 (0.85–0.90)	86%/98%	Clinicians 0.80; commercial 0.75	NPV 0.99; 73-feature SHAP interpretability
Herman 2024 (eOMI v1) [7]	OMI	2222 pt/2263 contacts	0.938 (0.924–0.951)	80.6%/93.7%	ECG experts 0.843; STEMI criteria 0.651	CE-marked; rescues 42–61% of OMI missed by experts/STEMI
Herman 2026 (aOMI/QoH) [20]	True STEMI + equivalents	1032	0.94 (0.92–0.95)	92.0%/81.0%	SoC sens 71.0%; FPA 41.8→7.9%	PPV 87.1%, NPV 87.9% at on-label threshold 0.5; accuracy 87.4%
Cho 2025 [21]	AMI req. revasc.	259,454 (external)	0.968 (0.965–0.971)	Sens 0.884/Spec 0.929 at Sens = 0.9 op point	—	ViT with SSL on 1.03 M ECGs; STEMI 0.991; NSTEMI 0.947
Wu 2022 (CNN-LSTM) [6]	STEMI	1857 ECG (external)	0.99 (Test 2)	94%/83%	Cardiologists 0.94	2-stage pipeline; culprit-vessel AUC 0.96 LAD; 0.81 RCA vs. LCx
Meyers 2021 (STDmaxV1-4 manual rule) [26]	Posterior OMI	808	n/a (binary rule)	37.4%/97.6%	Expert reader benchmark	LR+ 15.60; human-reader benchmark (NOT AI)

Abbreviations: AMI = acute myocardial infarction; AUC = area under the curve; CI = confidence interval; CNN-LSTM = convolutional neural network + long short-term memory; FPA = false-positive activation; NPV = negative predictive value; OMI = occlusion myocardial infarction; PPV = positive predictive value; QoH = Queen of Hearts; SHAP = Shapley additive explanations; SoC = standard of care; SSL = self-supervised learning; STEMI = ST-elevation myocardial infarction; ViT = vision transformer.

**Table 2:** Multimodal AI evidence across cardiac biomarkers, coronary CT angiography, invasive angiography, intracoronary imaging, and clinical risk prediction in ACS.

Modality	Representative Model/Trial	Cohort (*n*)	Key Metric	Strength	Limitation	Reference Standard	External Validation/Evidence Level
ML troponin	CoDE-ACS [16]	10,286 (ext.)	AUC 0.953; 61% rule-out	Continuous probability output	Cohort skew to high-resource settings	Adjudicated type 1 (or 4b/4c) MI (4th UDMI)	Yes (10,286 pts, 7 cohorts)
ML troponin (POC)	ARTEMIS-POC [28]	2560	Rule-out 35.1%; NPV 99.96%	Single POC measurement sufficient	Type 2 MI may be missed	Adjudicated type 1/2 MI (4th UDMI)	Yes (USA + Australia cohorts)
AI-CCTA stenosis	Kim 2025 (YOLOv4) [14]	676 (298 ext.)	Per-patient AUC 0.871; NPV 96.6%	Per-patient and per-artery analysis	Korean cohort, single-ethnicity	Obstructive CAD, ≥50% stenosis	Yes (298 pts, 3 external centers)
FFR-CT (NSTE-ACS)	Meier 2025 [37]	151	Vessel-level AUC 0.84 vs. 0.65 CCTA	Prospective, FFR-referenced	8% excluded for image quality	Invasive FFR	No (single trial)
AI-QCA	FLASH (Kim 2025) [12]	400 randomized (395 analyzed)	MSA non-inferior to OCT	First RCT vs. imaging reference	Predominantly stable/silent ischemia; higher malapposition 13.6% vs. 5.6%	Core-lab OCT (MSA)	Multicenter RCT (non-inferiority met)
Angiography-derived FFR (QFR, AngioPlus)	FAVOR III China [3]	3825	HR 0.65 (95% CI 0.51–0.83) for 1-year MACE vs. angiography	Large sham-controlled RCT; positive outcome	Stable/low-risk ACS; acute MI <72 h excluded; platform-specific (AngioPlus)	Angiography-guided PCI (not pressure-wire FFR)	2-year outcomes sustained; single QFR platform
Angiography-derived FFR (QFR, Medis)	FAVOR III Europe [4]	2000	HR 1.63 (95% CI 1.11–2.41) vs. FFR	Large randomized comparison	Did not meet non-inferiority; platform-specific	Pressure-wire FFR	Multicenter RCT (non-inferiority not met)
Angiography-derived FFR (FFRangio, CathWorks)	ALL-RISE [5]	1930	Non-inferior to FFR	International, 59 sites (North America, Europe, Asia, Middle East)	Excluded recent STEMI/NSTE-ACS culprits	Pressure-wire FFR	Multicenter RCT (non-inferiority met)
AI-OCT (plaque erosion)	Park 2022 (transformer) [11]	292 ACS (ext.)	Frame AUC 0.94; lesion 0.91	Transformer outperforms CNN	Single-vendor OCT cohort	Expert OCT (plaque erosion)	Yes (292 pts, independent external cohort)
AI-OCT (TCFA)	PECTUS-AI [8]	438 enrolled (414 analyzed)	Complete-pullback HR 5.50 (95% CI 1.94–15.62); NPV 97.6%	Prospective post-MI cohort; non-culprit-plaque, outcome-validated	European cohort; 2-year follow-up	Core-lab OCT + adjudicated 2-year MACE	No (single multicenter cohort)
AI-IVUS segmentation	DeepLabv3+ [52]	175 pullbacks	DSC 0.927 lumen/0.944 EEL	Multicenter dataset	No ACS-specific cohort	Core-lab manual lumen/EEL	No (single multicenter dataset)
ML bleeding prediction	PRAISE [50]	19,826 ACS + 3444 (ext.)	AUC 0.86 at 1 year (ext.)	BleeMACS + RENAMI multinational	Heterogeneous registry	BARC 3 or 5 bleeding	Yes (3444 pts external)
ML AKI prediction	Kuno 2022 (LightGBM) [10]	19,222	AUC 0.772	Only 7 SHAP-selected variables	Single-country (Japan) derivation	AKI (serum creatinine rise)	No (single-country)

Note: point estimates are shown; 95% confidence intervals are reported for hazard ratios. Negative predictive values are prevalence-dependent and reflect each cohort’s event rate. Abbreviations: ACS = acute coronary syndrome; AI = artificial intelligence; AKI = acute kidney injury; aAngioPlus = angiography-derived QFR system (Pulse Medical, Shanghai, China); ARTEMIS-POC = ML algorithm for point-of-care high-sensitivity cardiac troponin I rule-out; AUC = area under the ROC curve; BARC = Bleeding Academic Research Consortium; CAD = coronary artery disease; CCTA = coronary CT angiography; CI = confidence interval; CNN = convolutional neural network; CoDE-ACS = Collaboration for the Diagnosis and Evaluation of ACS; DSC = Dice similarity coefficient; EEL = external elastic lamina; ext. = external (validation cohort); FFR = fractional flow reserve; FFR-CT = FFR derived from CT; HR = hazard ratio; IVUS = intravascular ultrasound; MACE = major adverse cardiac events; MI = myocardial infarction; ML = machine learning; MSA = minimal stent area; NPV = negative predictive value; NSTE-ACS = non-ST-elevation acute coronary syndrome; OCT = optical coherence tomography; PECTUS-AI = AI thin-cap fibroatheroma detection study; POC = point-of-care; PRAISE = Prediction of Adverse Events in ACS; pts = patients; QCA = quantitative coronary angiography; QFR = quantitative flow ratio; RCT = randomized controlled trial; SHAP = Shapley additive explanations; STEMI = ST-elevation myocardial infarction; TCFA = thin-cap fibroatheroma; UDMI = Universal Definition of Myocardial Infarction.

**Table 3:** Implementation considerations for AI in ACS care.

Domain	Current State	Key Constraint	Operational Response
Regulatory clearance	CE-mark for PMcardio (eOMI/aOMI), FDA Breakthrough Designation 2025; HeartFlow FFR-CT (FDA-cleared 2014); Ultreon 3.0 (FDA + CE, 2026) [62]	No FDA-cleared AI-ECG for OMI; FFR-angio platform-specific evidence only	Pre-market conformity assessment under EU AI Act; FDA SaMD predetermined change control plan
External validation	14.7–23.9% of ICU AI scoring systems externally validated; mean AUC decline 0.037 on independent testing—analogy applied to cardiovascular AI	Population, practice, and data drift over time	Continuous local re-validation; longitudinal AI surveillance with automated alerts
Algorithmic equity	Training sets dominated by North American, European, East Asian cohorts	Sex-specific calibration incomplete; under-representation of S Asian, African, Latin American populations	Mandate diverse cohort representation in AI validation; federated learning across geographies
Cost and reimbursement	No CMS/NICE cost-effectiveness consensus for most AI tools	Licensing tens to hundreds of thousands of dollars per year per institution	Health-economic analyses tied to outcome trials; transparent ICER reporting

Abbreviations: CMS = Centers for Medicare & Medicaid Services; FDA = Food and Drug Administration; ICER = incremental cost-effectiveness ratio; NICE = National Institute for Health and Care Excellence; SaMD = software as a medical device.

## Data Availability

All data discussed in this review are available in the cited primary publications. The verified reference workbook supporting Section 3 (AI_ECG_MASTER v4.1, 18 April 2026) is available from the corresponding author upon reasonable request.

## Abbreviations

ACC	American College of Cardiology
ACS	Acute coronary syndrome
AHA	American Heart Association
AI	Artificial intelligence
AI-QCA	Artificial intelligence quantitative coronary angiography
AKI	Acute kidney injury
AMI	Acute myocardial infarction
AUC	Area under the (receiver operating characteristic) curve
CABG	Coronary artery bypass grafting
CAD-RADS	Coronary artery disease reporting and data system
CCTA	Coronary computed tomography angiography
CI	Confidence interval
CNN	Convolutional neural network
CoDE-ACS	Collaboration for the Diagnosis and Evaluation of Acute Coronary Syndrome
DAPT	Dual antiplatelet therapy
DCR	Dynamic coronary roadmap
DICOM	Digital Imaging and Communications in Medicine
ECG	Electrocardiogram/electrocardiography
ED	Emergency department
EEL	External elastic lamina
eGFR	Estimated glomerular filtration rate
ESC	European Society of Cardiology
FDA	Food and Drug Administration
FFR	Fractional flow reserve
FFR-CT	Fractional flow reserve from coronary CT
FPA	False-positive activation
GRACE	Global Registry of Acute Coronary Events
GPU	Graphics processing unit
HD-IVUS	High-definition intravascular ultrasound
HR	Hazard ratio
hs-cTnI	High-sensitivity cardiac troponin I
hs-cTnT	High-sensitivity cardiac troponin T
IVUS	Intravascular ultrasound
LAD	Left anterior descending (artery)
LCx	Left circumflex (artery)
LCR	Lipid-to-cap ratio
LightGBM	Light gradient boosting machine
LSTM	Long short-term memory
MACE	Major adverse cardiovascular events
MI	Myocardial infarction
MI3	Myocardial ischemic injury index
ML	Machine learning
MSA	Minimal stent area
NCDR	National Cardiovascular Data Registry
NPV	Negative predictive value
NSTE-ACS	Non-ST-elevation acute coronary syndrome
NSTEMI	Non-ST-elevation myocardial infarction
OCT	Optical coherence tomography
OFR	Optical flow ratio
OMI	Occlusion myocardial infarction
PCAT	Pericoronary adipose tissue (FAI: fat attenuation index)
PCI	Percutaneous coronary intervention
PPV	Positive predictive value
QCA	Quantitative coronary angiography
QFR	Quantitative flow ratio
RCA	Right coronary artery
RCT	Randomized controlled trial
ResNet	Residual neural network
SaMD	Software as a medical device
SHAP	Shapley additive explanations
STEMI	ST-elevation myocardial infarction
TCFA	Thin-cap fibroatheroma
ViT	Vision transformer
XGBoost	Extreme gradient boosting
